# Mechanical Properties in the Glioma Microenvironment: Emerging Insights and Theranostic Opportunities

**DOI:** 10.3389/fonc.2021.805628

**Published:** 2022-01-21

**Authors:** Adip G. Bhargav, Joseph S. Domino, Roukoz Chamoun, Sufi M. Thomas

**Affiliations:** ^1^ Department of Neurological Surgery, University of Kansas Medical Center, Kansas City, KS, United States; ^2^ Department of Otolaryngology, University of Kansas Medical Center, Kansas City, KS, United States

**Keywords:** glioma, heterogeneity, tumor microenvironment, biophysical properties, tissue mechanics

## Abstract

Gliomas represent the most common malignant primary brain tumors, and a high-grade subset of these tumors including glioblastoma are particularly refractory to current standard-of-care therapies including maximal surgical resection and chemoradiation. The prognosis of patients with these tumors continues to be poor with existing treatments and understanding treatment failure is required. The dynamic interplay between the tumor and its microenvironment has been increasingly recognized as a key mechanism by which cellular adaptation, tumor heterogeneity, and treatment resistance develops. Beyond ongoing lines of investigation into the peritumoral cellular milieu and microenvironmental architecture, recent studies have identified the growing role of mechanical properties of the microenvironment. Elucidating the impact of these biophysical factors on disease heterogeneity is crucial for designing durable therapies and may offer novel approaches for intervention and disease monitoring. Specifically, pharmacologic targeting of mechanical signal transduction substrates such as specific ion channels that have been implicated in glioma progression or the development of agents that alter the mechanical properties of the microenvironment to halt disease progression have the potential to be promising treatment strategies based on early studies. Similarly, the development of technology to measure mechanical properties of the microenvironment *in vitro* and *in vivo* and simulate these properties in bioengineered models may facilitate the use of mechanical properties as diagnostic or prognostic biomarkers that can guide treatment. Here, we review current perspectives on the influence of mechanical properties in glioma with a focus on biophysical features of tumor-adjacent tissue, the role of fluid mechanics, and mechanisms of mechanical signal transduction. We highlight the implications of recent discoveries for novel diagnostics, therapeutic targets, and accurate preclinical modeling of glioma.

## Introduction

### Contemporary Management of Malignant Glioma and Biological Considerations

Mortality due to cancer continues to rise worldwide with improving medical management of other disease processes. Brain cancer, specifically, represents one of the most lethal cancer subtypes. Malignant gliomas are a group of primary brain tumors that harbor a poor prognosis for afflicted patients (1, 2). Though there is some variation in survival rates ranging from months to decades among the different histological and molecular categories and grades of gliomas, in general, current therapies are not curative. Within this group is a subset of particularly high-grade tumors including glioblastoma which portend the worst survival with recent estimates of median survival at 8 to 14 months and a 7.2% 5-year survival rate post-diagnosis (1, 3). Unfortunately, this group also comprises the most common type of malignant glioma accounting for approximately 48.6% of all primary malignant brain tumors (1).

The standard of care and outcomes for glioblastoma have been largely unchanged since the development of the Stupp protocol (4, 5). Contemporary management of glioblastoma aims for maximal cytoreductive surgery while preserving critical neurologic function that is followed by adjunctive chemotherapy with temozolomide and fractionated radiotherapy (6). In certain cases, this treatment algorithm is limited by patient and disease factors including fitness to undergo aggressive therapy and tumor location, respectively. In such cases, intervention is directed towards establishing a definitive diagnosis as with biopsy and mitigating symptoms (6).

In light of poor outcomes in patients with glioblastoma, several lines of investigation are ongoing in order to develop novel therapeutics and treatment strategies (7–12). Importantly, advances in the understanding of tumor biology and influences of the microenvironment have begun to inform emerging paradigms for management of glioma. Glioma stem cells or brain tumor-initiating cells (BTICs) have been established as a subset of cells within glioma that contribute to treatment resistance and recurrence of disease. These cells exhibit properties including chemoresistance and radioresistance as well as considerable heterogeneity and plasticity on multiple levels which has posed a therapeutic challenge (13–18). Heterogeneity of BTICs encompasses variation in tumor characteristics over time—temporal heterogeneity, variation in tumor and cellular characteristics depending on location within a tumor—locoregional heterogeneity, and variation in disease characteristics from patient to patient—population heterogeneity which can impact response to treatment. As a result, current investigations are transitioning from single-agent or single-target therapies to treatment modalities with robust mechanisms of action that may overcome disease heterogeneity. Similarly, robust mechanisms of action are required to bypass tumor plasticity and changes in response to unimodal therapies (8, 17, 19–21). Examples of such efforts include cell-based therapies, immunotherapy approaches, and bioengineering strategies such as gene therapy, and excellent overviews of these topics can be found elsewhere (8, 9, 11, 22–24).

Improved understanding of plasticity and heterogeneity of BTICs has also led to further study of the tumor microenvironment and its inherent heterogeneity and plasticity. Similar to BTICs, studies have demonstrated that cellular and vascular components of the microenvironment respond to tumorigenesis and treatment in ways that may be facilitating malignant adaptation in tumor cells (16, 25–27). The potential bidirectional interaction between tumor cells and tumor microenvironment is particularly evident when considering the influence of immune-active cells such as myeloid-derived suppressor cells on immunosuppression *via* immune cell dysregulation (16, 17, 25, 28). Although cellular and vascular niches in the tumor microenvironment are now known to be a key player in the tumor milieu, the study of the mechanical properties of the microenvironment is relatively new. As with other components of the microenvironment, evidence suggests an important evolving role for mechanical properties in the context of treatment resistance and disease.

### Tissue Mechanics in the Brain

Tissue mechanics broadly comprise cellular and tissue stiffness properties as well as stresses transmitted by fluid including cerebrospinal fluid (CSF) dynamics and interstitial fluid pressures. The influence of tissue mechanics on normal brain development and homeostasis has been well-described (29, 30). In the developing brain, stiffness gradients arise during various stages of embryogenesis and migration of neural precursors and neural stem cell populations particularly in the subventricular zones of the brain. Gradients have been attributed to maturation of cellular cytoarchitecture and changes in extracellular matrix (ECM) composition that facilitate normal migratory and tissue organization in development (31–34). As a result, a general trend towards increased global brain stiffness is seen with notable regions of ‘softer’ brain such as the hippocampus in the adult brain (35–38). In addition to spatial organization of cells and tissue, heterogeneity of tissue mechanics in non-disease states is important for directing differentiation and cell-type determination of embryonic neural stem cells as well as adult stem cells. Notably, aberrant mechanical signaling from the microenvironment has tremendous implications for regulating the behavior and plasticity of BTICs and preclinical models of BTICs that is discussed in detail in subsequent sections (29, 30).

### Tissue Mechanics in Disease and Cancer

Alteration of the inherent spatiotemporal heterogeneity during development and maturation contributes to various disease states including traumatic brain injury, neurodegenerative diseases, and cancer (29, 39, 40). Briefly, studies demonstrate a progressive loss of global brain stiffness in the context of neurodegenerative disease that is contrary to generalized stiffening of the brain in aging, and this is secondary to cellular injury and compromise of cell-intrinsic mechanical factors as well as cell-extrinsic factors such as breakdown of the basement membrane in certain disease processes and changes in the composition of the ECM (29, 41–44). Conversely, preliminary studies demonstrate elevation in pro-stiffening factors such as Tenascin-C (TNC) in the setting of traumatic brain injury with evidence suggesting enhancement of mechanical signaling likely owing to changes in intracranial pressure, injury from intracranial hemorrhage, and direct injury to areas of adult neurogenesis that may affect long-term outcomes and neurodegeneration (29, 39, 40).

The study of physical traits and microenvironmental mechanics in cancer is a relatively young field compared to research surrounding the traditional hallmarks of cancer. In recent years, the importance of physical characteristics in addition to biological factors has been increasingly recognized and extensively investigated in several cancers including breast cancer and carcinomas of the gastrointestinal system. From this work, ECM stiffness has emerged as a physical hallmark of many cancers that contributes to tumorigenesis, metastasis, metabolism, immune response and numerous additional processes (45–50). Though discussion of tissue mechanics in other cancer types is beyond the scope of this review, we highlight important principles gleaned from work in other cancers that may offer a template for further investigation of tissue mechanics in glioma, which is comparatively in its early stages. Comprehensive reviews of these fundamental discoveries and the work leading to these findings can be found elsewhere (46, 47, 51, 52). Investigation of tissue mechanics of the cancer microenvironment in various models has illuminated three biophysical concepts: 1) solid stress 2) fluid stress and 3) stiffness. Solid stress refers to amount of force per area present in the region of interest whether it is the tumor or the surrounding milieu (52, 51). The key factor influencing solid stress is derived from tumor tissue and cellular properties, though there is also contribution from ECM and surrounding components. Increased proliferation of cells within a tumor transmits increased stress through the space-limited tumor microenvironment. In addition, spatial and geometric considerations can also exacerbate regional solid stress based on the alignment of cellular cytoskeletal components relative to ECM matrix components as well as through a mechanism referred to as “jamming” whereby cumulative stress is increased after a critical cell population is reached that augments cell to cell contact and force (46, 51, 52). Fluid stress is the result of perturbation of interstitial fluid pressure as well as shear flow in certain microenvironments such as adjacent to the ventricles of the brain. Leaky tumor vasculature, impaired lymphatic drainage, and vascular compression secondary to solid stress can all contribute to increased interstitial pressure (51, 52).Global elevation in fluid stress as in the case of increased intracranial pressure in the fixed volume of the cranium can also exacerbate fluid stress at the tissue level. Finally, stiffness refers to the resistance to deformation as a result of stress and can be used to describe the tumor as a whole, individual cells, or the microenvironment and its components including the ECM. Global tissue stiffness is affected by ECM deposition or degradation, ECM cross-linking and changes in microarchitecture, and at the cellular level by cytoskeletal remodeling and cell contraction (45, 51, 52).

Interdependence between certain hallmarks of cancer and physical traits in the microenvironment is a relatively unexplored area in brain cancer but has been described in other cancer types (47–49, 52). Recent studies have revealed a potential link between immune escape and tissue mechanics where regional stress may impair vascular and lymphatic channels (49, 51, 52). This results in reduced migration of immune effector cells to the tumor site and effectively creates a functional immune escape phenomenon. Relatedly, cellular deformation secondary to solid and fluid stress may affect the integrity of intracellular structures including the nucleus and alter expression of immune soluble factors through direct physical perturbation as well as through mechanisms of mechanotransduction that affect downstream genetic and epigenetic regulation (49, 51, 52). A similar type of interdependence is observed with tumor metabolism and tissue mechanics; it is possible that solid and fluid stress may significantly predispose tumor cells towards aberrant metabolism in a feed-forward mechanism that continues to progress as the tumor grows and microenvironmental stress increases. One example of this is the Warburg effect and the interplay between stress and aerobic glycolysis: increased regional stress may promote hypoxia *via* vascular compression within the tumor and thereby apply selection towards aerobic glycolysis to facilitate tumor growth and progression (48). Overarching these overlapping mechanisms is the concept of mechanoreciprocity which mirrors the dynamic seen between cancer cells and the biological factors of the microenvironment such as the dynamic interactions between BTICs and immune cells or BTICs and neurons in the tumor milieu (17, 46). In the same way, the interaction between cancer cell and physical properties of the microenvironment is also dynamic and has been most extensively documented in the process of cancer migration where reciprocal signal transduction and physical changes at the cellular level and tissue level enable the requisite cellular geometric changes, elasticity, and focal adhesions to achieve metastasis (47, 51, 52). This dynamic interaction acts as the substrate for heterogeneity and plasticity in the physical traits of the tumor and microenvironment much like biological receptor- or soluble factor-mediated cell communication and therapy-induced changes to the cellular phenotype or genetics.

## Biophysical Features of Glioma-Adjacent Tissue

### Current Understanding of Tissue Mechanics in the Glioma Microenvironment

**Figure 1:**
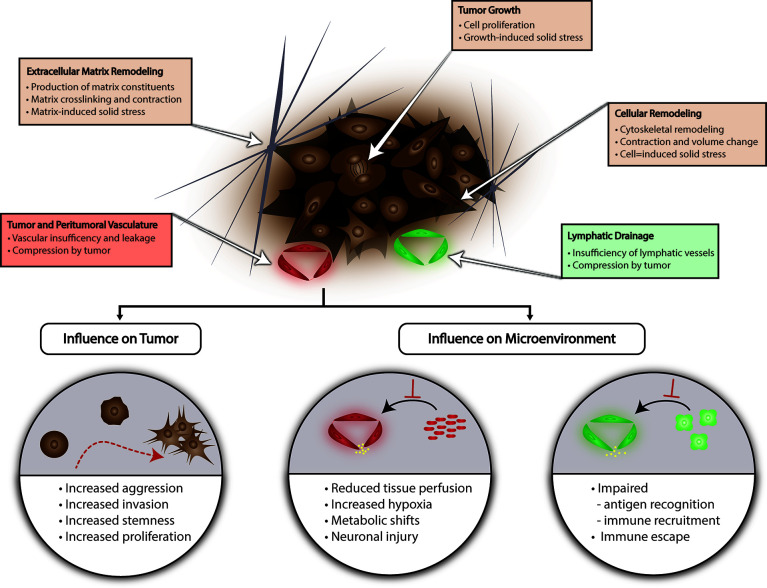
Tissue Mechanics in Glioma. Several factors contribute to generation of solid stress in the glioma microenvironment including lymphatic or vascular insufficiency, tumor growth, and ECM or cellular remodeling. Solid stress can promote glioma aggression and create a microenvironment conducive for immune escape and BTIC selection. ECM, extracellular matrix; BTIC, brain tumor-initiating cell.

Tissue mechanics in the glioma microenvironment primarily refers to solid stress from contributions by the surrounding ECM and tissue architecture as well as the cellular compartment in the tumor milieu which includes glioma cells. Unlike many other systemic cancers where the causative factors of solid stress may be more intuitive owing to typical growth patterns characterized by displacement of surrounding tissue *via* mass effect, gliomas tend to exhibit an infiltrative growth pattern. The components of physical stress generation in the microenvironment were unknown until recently. These are summarized in **Figure 1**. Stylianopoulos et al. developed a mathematical model to calculate growth-induced solid stress in a tumor by measuring the extent of deformation of tumor as stress is released from the tumor microenvironment after a cut is made along the long axis of the tumor (53). Various orthotopic cancer lines were employed to assess tumor-related solid stress and notably the U87 human glioblastoma cell line. Briefly, inoculation of tumor was performed in the flank of immunocompromised mice, and tumor was excised after reaching a tumor size of 1cm^3^. The solid stress-release assay was performed in different iterations after treatment with agents to selectively deplete individual components of the tumor microenvironment and thereby identify the contributory factors to solid stress. Selective depletion of U87 cancer cells or collagen in the ECM produced almost a two-fold decrease in tumor opening, a surrogate measurement for solid stress compared to control tumor. Importantly, no relationship between interstitial fluid pressure and solid stress was noted suggesting that cellular and ECM factors are the primary contributors (53). Nia et al. later developed an alternate method to characterize growth-induced solid stress *in situ* as well as *ex vivo* measurement (54). For *ex vivo* measurement, following tumor excision in the manner aforementioned, tumor was embedded in agarose gel. A planar cut is then performed, and the 2-dimensional planar deformation of the tumor is measured using an ultrasound probe. *In situ* assessment of solid stress in a murine orthotopic model of U87 GBM was subsequently performed by using a cylindrical punch to excavate a component of the tumor through the cranial window and measuring deformation with ultrasound. Interestingly, initial *ex vivo* 2-dimensional mapping of embedded U87 GBM demonstrated greater compressive stress at the periphery compared to the central core. Additionally, these tumors experienced less solid stress compared to other tumor types (maximum stress of 0.21 kPa versus 7 kPa in pancreatic tumors) (54). *In situ* measurement of solid stress revealed a significant influence of the surrounding tumor microenvironment—the cranial vault with fixed volume and surrounding normal tissue. Lesser degree of deformation was observed *ex vivo* compared to *in situ* measurement and approximately 0.02 kPa and 0.1 kPa compressive forces, respectively (54). This finding confirms the importance of considering the cranial vault as a fixed volume and accounting for the consequences when examining tissue mechanics in brain tumors. For example, states with increased intracranial pressure due to ancillary causes such as obstructive hydrocephalus may impact glioma microenvironment solid stress. Stylianopoulos et al. utilizing the previously described tumor relaxation model and mathematical model that the amount of deformation in U87 GBM tumors is proportional to the stored solid stress in the tumor microenvironment, and that solid stress forces in the periphery contribute to vascular and lymphatic collapse (55). This represents a possible mechanism based in the mechanical properties of the tumor microenvironment for BTIC selection and plasticity; impaired perfusion and lymphatic drainage may promote hypoxia within the tumor and a milieu conducive for aggressive, resistant cellular phenotypes as well as secondarily creating a physical barrier for potential cellular therapy or conventional chemotherapy **(** **Figure 1** **)**. Seano and Nia et al. relate tumor growth pattern to the amount of solid stress imposed on surrounding normal brain tissue using *in situ*, ultrasound-based measurement of stress (56). They utilize two distinct GBM cell lines U87 and MGG8 which exhibit nodular and infiltrative growth, respectively in an orthotopic murine model. Analysis of solid stress in these groups revealed qualitative deformation of surrounding tissue on histology with U87 nodular tumors, but not MGG8 tumors. This was corroborated with stress measurements that showed lesser radial and circumferential stress in the MGG8 infiltrative tumors compared to U87 nodular tumors (MGG8: radial stress 0.014 +/- 0.001 kPa, circumferential stress 0.063 +/- 0.004 kPa; U87: radial stress 0.020 +/- 0.001 kPa, circumferential stress 0.110 +/- 0.005 kPa) (56).

Although the fundamental mechanisms of growth-induced solid stress and the variables affecting this has been elucidated over the past decade, investigating these features in humans within a complex biophysical system continues to be a challenge. In large part due to limitations of current technologies, noninvasive methods of assessing physical properties of intracranial tumors are still being optimized by several groups (57–61). Initial studies have begun to define the biophysical properties of glioma, albeit with some discrepancy among groups. Specifically, the stiffness of glioma tissue and the microenvironment is debated, contrary to other systemic cancers where tumor stiffness is a hallmark. Schregel et al. characterized physical parameters of orthotopic G30 BTIC cell line using magnetic resonance elastography (MRE) (62). Analysis of MRE parameters revealed significant heterogeneity within tumor tissue as indicated by viscoelastic modulus and shear wave speed; stiffer areas on MRE corresponded to regions of high cell density on histology, and softer areas corresponded to regions necrosis and lower cell density (62). *In vivo*, intraoperative MRE of brain tumors performed by some groups suggested increased stiffness of brain tumor compared to normal brain as well as a trend of increasing stiffness with lower grade tumors (57, 58). Notably, these were the only studies to utilize MRE intraoperatively. Chauvet et al. report young’s modulus ranging from 11.4 kPa to 33.1 kPa by shear wave elastography including meningiomas, low-grade gliomas, high-grade gliomas, and metastases. Meningiomas exhibited the greatest stiffness (33.1 +/- 5.9 kPa) whereas high-grade gliomas exhibited the least stiffness (11.4 +/- 4.9 kPa); however, tumors on average were stiffer than normal brain stiffness of 7.3 +/- 3.6 kPa (58). Other groups report similar trends with tumor grade, but report glioma tissue as softer than normal brain tissue though there is a small subset of gliomas that are stiffer in these studies (59–61). Reiss-Zimmerman et al. observe no significant difference in the elasticity parameter with MRE among tumor types whereas the elasticity component reflects the trend reported in the other studies—meningiomas exhibiting the least elasticity likely owing to higher cellular density compared to infiltrative gliomas. In this study, average young’s modulus for all tumors was 1.43 +/- 0.33 kPa while the average for normal white matter was 1.62 +/- 0.27 (59). Moreover, intertumoral heterogeneity and intratumoral heterogeneity in viscoelastic properties were evident among gliomas as well as meningiomas to a lesser degree and may be due to high rates of cell turnover and heterogeneous distribution of cell density in gliomas (59).

The biological substrate of tissue stiffness also requires special consideration in the context of gliomas. Typical constituents of the ECM found elsewhere in the body are absent in the brain, namely collagen, fibronectin, and laminin (63–65). Instead, proteoglycans as well as heparin sulfate and hyaluronic acid are present. Proteins involved in cellular adhesion, particularly TNC, are also present and these constituents are frequently enriched in gliomas which can contribute to ECM stiffness **(** 63–65 **).** The orchestration of changes in glioma microenvironment stiffness and the subsequent mechano-transduction remains unclear. Although aberrant production of ECM constituents, even in the absence of typical proteins such as collagen in the brain, likely plays a role in promoting glioma tissue stiffness, other mechanisms have also been described. Pogoda et al. report a phenomenon of compression stiffening in glioma tissue obtained from biopsy specimens of patients with GBM (66). They demonstrate that the young’s moduli measured in patient GBM tissue is not significantly different from the viscoelasticity of normal mouse brain tissue which was used as a proxy for normal human brain. With the addition of compressive force, the relative young’s modulus for GBM tissue compared to normal brain tissue increased significantly with increasing compressive force to a relative young’s modulus of approximately 1.8 at 20% compression of tissue (66). Additionally, using the LN229 GBM cell line, Pogoda et al. showed cell stiffness and morphology was dependent on substrate stiffness which was assessed by gel cultures of varying stiffness (66). Compared to normal astrocytes, a monotonic dependence of cell adherent area on substrate shear modulus ranging from 300 to 14,000 Pa was observed. A similar relationship was observed with cell stiffness with a maximal cell stiffness of approximately 5 kPa achieved at 14 kPa of substrate stiffness after which no further changes in cellular stiffness was observed (66). Taken together, these findings suggests that although glioma tissue may be softer at baseline compared to surrounding tissue, regional normal brain stiffness can stimulate local changes in glioma stiffness signifying cytoskeletal remodeling and motility. A compression-driven mechanism of focal glioma stiffness may complement previously recognized mechanisms of primary ECM stiffening due to changes in ECM composition and deposition which is likely not the sole mediator of glioma stiffness. Compression from surrounding normal brain tissue can arise from various processes including increased intracranial pressure or interstitial pressures which are not uncommon in intracranial malignancy.

A recent MRE-based study and mathematical analysis by Streitberger et al. offer further reconciliation of relative glioma “softness” compared to normal brain and tumor progression and invasion (67). The authors first devised a phantom model mimicking the ECM and cytoarchitecture of meningioma and glioma using amalgamations of agar, heparin, and tofu. Viscoelastic properties were measured using MRE at varying solid composition ratios of the model and varying water content to determine how the viscoelastic properties of an intracranial tumor may change in response to increased fluid due to disrupted blood-brain barrier in surrounding vasculature (67). In their phantom models, different substrates functionally recapitulated different components of glioma ECM—abundant glucosaminoglycans (GAGs), absence of significant fibrosis in the case of heparin while the tofu phantom mimicked an entanglement of proteins governed by hydrophobic interactions with the high collagen content observed in meningioma with relatively high viscosity and increased fluidity (67). With glioma and the heparin phantom model, an inverse relationship between water absorption and viscosity was observed (67). Indeed, this was corroborated with MRE imaging studies in patients where T2 signal reflected higher water content and this corresponded to lower viscosity, i.e. lower phase angle and consequently decreased fluidity. Interestingly, other groups have also shown that increased ADC signal in GBM corresponding to higher water content may be associated with worse outcomes (68, 69). Based on these findings, the authors posit that GBM behaves functionally as a low viscosity, low fluidity solid secondary to the ability of significant, hydrophilic GAGs in the ECM to bind to water without meaningful perturbation of viscosity (67). On the other hand, meningioma behaves like a high viscosity, high fluidity fluid with ECM consisting of entangled proteins with hydrophobic aggregation. In the case of meningioma, the variation in focal shear stress can steeply increase stiffness due to local drainage of water followed by direct solid-solid contact of ECM constituents and protein coagulation (67). With regards to glioma infiltrative growth and tumor progression, viscous fingering. Briefly, in viscous fingering, the less viscous glioma with lower regional surface tension is pushed into the higher viscosity surrounding producing the characteristic microinvasive infiltration without clear margins (67). Overall, these studies suggest a unifying theory though additional work is required to elucidate the impact of tumor subtype, tumor location such as proximity to the ventricle, tumor/microenvironment heterogeneity, among other factors.

### Influence of Microenvironmental Tissue Mechanics on Glioma Malignancy

Though studies examining the impact of tissue mechanics are limited, this is an area of considerable interest and active research with themes beginning to emerge. Unsurprisingly, given discoveries in tissue mechanics in other types of cancers, tissue mechanics in glioma tend to regulate hallmarks of cancer. Here we highlight representative studies that have uncovered these mechanisms **(** **Figure 1** **)**.

Several studies have linked alterations in microenvironmental rigidity to enhanced glioma aggression, and some have also identified certain microenvironmental features as prognostic markers for survival at the patient level (70–73). Miroshnikova et al. established the importance of HIF1α signaling, IDH status, and ECM components in GBM (70). Using *in vitro* models as well as human tumor samples, the authors show that increasing in ECM stiffness is observed with increasing grade of tumor in glioma ranging from 50-1,400 Pa in low grade gliomas to 70-13,500 in higher grade gliomas; analysis of human samples revealed worst patient prognosis with tumors with a high proportion of stiff ECM >1,400 Pa (70). Mechanistically, they define TNC and hyaluronic acid (HA) as key contributors to ECM stiffness in glioma and show increased levels of these constituents is associated with worse survival and stiffer tumor ECM. Finally, they describe the downstream effects of HIF1α expression in a hypoxic tumor microenvironment (70). HIF1α serves as a regulator of TNC expression and ultimately glioma microenvironment mechanical properties through stiffening of the ECM. At the tumor level, the authors show that perturbation of TNC-mediated ECM stiffening or at a point in the regulatory pathway with HIF1α or one of its regulators miR-203 improved survival is observed in murine xenograft models of GBM. Notably, wild-type IDH *via* onco-metabolite (R)-2-hydroxyglutarate is implicated in HIF1α regulation and consequently ECM stiffness regulation; in patients with recurrent IDH-mutant tumor, ECM stiffness was noted to increase comparing initial diagnosis and associated increased TNC expression suggesting a mechanosignaling-induced tumor aggression (70). Chen et al. similarly define a key mechanosignaling axis involving the PIEZO1 ion channel that has been shown to be overexpressed in a variety of cancers including all subtypes and grades of glioma signifying a potential common, evolutionarily conserved mechanosensation mechanism in cancer (71). Beginning with a *Drosophila* model of glioma and subsequently utilizing GBM and BTIC cell lines, the authors demonstrated that the PIEZO1 channel is necessary for tumor growth *in vitro and in vivo* and abrogation of the ion channel resulted in significantly longer survival and reduced tumor growth in mouse models (71). Further characterization of PIEZO1 as a central hub in a mechanotransduction cascade revealed subcellular location of the channel at sites of focal adhesion and a feed-forward mechanism whereby PIEZO1 was shown to be essential for ECM stiffening *via* regulation of other ECM remodeling genes including TAZ and FHL3 as well as glioma cell mechanotransduction (71). In experiments with PIEZO1 knockdown, stiffness-dependent glioma cell growth was not observed, and in experiments assessing the stiffness-dependent growth of glioma cells in response to varying stiffness hydrogels, increased expression of PIEZO1 was observed (71). Other groups have also confirmed the influence of microenvironmental stiffness on glioma growth and proliferation in the context of other signaling pathways including the EGFR and Rho/GTPase signaling pathways (72–74).

In addition to promoting glioma growth and proliferation, mechanical cues from the microenvironment also enhance other aspects of glioma stemness, specifically migration and invasion which enables diffuse spread through brain parenchyma (75–77). Zhang et al. elucidate a mechanism involving the cytokine IL-33 and receptor ST2 in the tumor milieu that stimulates expression and accumulation of TNC *via* NF-κB signaling in the microenvironment (76). Using a transwell migration assay, they demonstrate that IL-33 treatment produces a nearly 3-fold increase in invaded cells in a TNC-dependent matter; the authors observed enhanced migration on *in vivo* histologic analysis as well (76). Kim et al. identify another mechanosensation mediator in the CD44-HA interaction that also enhances glioma cell invasion (77). Aberrant and increased expression of CD44 and HA is present in the glioma microenvironment, and Kim et al. show that this ligand-mediated interaction is distinct from other adhesion interactions as with integrins and is stiffness-dependent (77). Using U373-MG glioma cells in transwell invasion assay and time-lapse microscopy, the authors show that the migration speed and invasion properties of these cells in the presence of CD44/HA improves with increasing stiffness of the HA hydrogel in which they are cultured (77). Integrin-mediated interaction between glioma cells and microenvironment components such as the glycocalyx or BCL9L have also been described and shown to enhance glioma stemness (75, 78, 79). Notably, in these studies, glioma stemness also incorporates the treatment-resistant quality of this phenotype and the authors show treatment sensitization employing gain- or loss-of-function methodologies to disturb the specific integrin-mediated mechanosensation (78, 79). Barnes et al. specifically examine recurrent GBM and determine that a bulky glycocalyx in the tumor microenvironment, that is frequently seen in recurrent GBM, interacts with integrins with mechanoreciprocity whereby downstream signaling enhances stemness of the cell which in turn effects increased tension in the glycocalyx to form a feedback loop (79).

Interestingly, in investigations by Miroshnikova et al. and others, GBM tumor tissue was observed to be stiffer than normal tissue and a trend was observed towards increased stiffness with high grade; in contrast, previously discussed studies found gliomas to be softer compared to normal brain and to exhibit the opposite trend with grade (59, 60, 66, 67, 70, 78). This underscores the complexity of measuring mechanical properties of the glioma microenvironments and requires further study to elucidate with consideration of standardized measurement techniques and preclinical modeling. Moreover, this may also be an indicator of the challenge associated with defining features of a heterogeneous disease process including sampling bias of the tumor as well as other confounders such as patient comorbidities and history of treatment.

Microenvironmental tissue properties also induce changes in tumor-adjacent tissue compartments in addition to glioma cells **(** **Figure 1** **)**. Seano and Nia et al. show that glioma tumor growth in an orthotopic murine model compresses tumor-adjacent vasculature and decreases perfusion (56). They note that this effect is more pronounced in glioma cell lines characteristic for nodular growth pattern—U87, GL261, and BT474 compared with cell lines that exhibit infiltrative growth. For example, at a 20-day timepoint, the authors demonstrate that the nodular cell line U87 resulted in a significantly reduced intravital perfused blood volume fraction compared to baseline (0.35 to 0.2) in surrounding vasculature whereas no significant changes were observed in the infiltrative cell line MGG8 in the same time frame (56). In tumors models with nodular growth, perfusion in the surrounding vasculature is also inversely correlated with tumor growth (56). MRI-based analysis of perfusion in tumor adjacent regions, not including peritumoral T2/FLAIR signal in a cohort of patients with GBM confirmed reduced perfusion in up to 53% of patients (56). Histological and behavioral analysis after tumor-related brain compression in the intracranial tumor mouse models also revealed evidence of neuronal injury and neuroinflammation as well as concordant significant changes in locomotion and gait suggesting deleterious neuronal sequelae from the physical effects of tumor growth (56). Tumor-related mechanical changes in the microenvironment can also promote local immune dysfunction. This type of microenvironment-mediated immunosuppression has been characterized in a variety of cancers and may result from tumor-related solid stress transmitted through the microenvironment as well as soluble factors and interactions with tumor-associated vasculature (80). In glioma specifically, Huang et al. show GBM ECM-based inhibition of T cell migration into the tumor milieu. Furthermore, they define an inverse correlation between TNC expression in the ECM and T cell transmigration (81). Increased levels of TNC *in vivo* in a mouse model of GBM was associated with reduced T cell enrichment in tumor tissue on histologic analysis, and assessment of *in vivo* transmigration using a mouse air pouch model demonstrated TNC-mediated transmigration of T cells (81). Pathway analysis *in vitro* using co-cultures of Jurkat cells with U118MG glioma tumor cells or tumor ECM identified phosphorylation of focal adhesion kinase (FAK) and migration-related kinase ERK which was shown to be required for transmigration through a cancer monolayer (81). Interestingly, a potential relationship between tumor ECM constituents and peritumoral edema was also observed in a study by Qu et al. analyzing the expression of GBM PIEZO1 relative to normal peritumoral tissue in patients; quantification of PIEZO1 expression and image analysis revealed a positive relationship between expression and extent of peritumoral edema where higher expression was observed in patients with severe edema that was defined as an edema index >3 (calculated as the ratio of tumor and edema volume to tumor volume) (82). Lastly, it is well known that GBM and the tumor microenvironment present numerous challenges to adequate and effective delivery of therapeutics, and this concept also extends to the physical properties of the tumor and microenvironment. Recent efforts have included innovative strategies to create therapeutics that account for and accommodate the rheological features to engineer adaptive therapeutics (83). Detailed overview of recent trends and advances in overcoming physical barriers to drug delivery can be found elsewhere (84).

### Mechanisms of Mechanical Stimuli Transduction in Glioma

Mechanistic understanding of mechanical stimuli transduction in glioma is lacking, though recent advances have shed some light on the microenvironment and cellular network interactions that underlie the effects of mechanical properties on disease progression. Hubs of mechanical stimuli transduction in gliomas can be generally categorized as either *via* mechanosensitive ion channels or non-ion channel-based mechanotransduction which encompasses a complex swath of poorly understood signaling pathways including integrin signaling, ligand-mediated signaling through interaction with ECM components, or receptor-mediated signaling through interaction with ECM components (85). Here, we briefly summarize representative mechanisms in the evolving framework of glioma mechanotransduction **(** **Table 1** **)**.

**Table 1:** Representative Substrates of Mechanical Signal Transduction in Glioma.

Class	Substrate	Effect	Model	Ref.
*Mechanosensitive Ion Channel*	PIEZO1	Promotes glioma aggression, growth; reduces survival *in vivo*	Murine, Xenograft	(71)
	TRP1	Cell migration, chemotaxis	Cell Culture	(86)
	ENaC	Cell volume regulation	Cell Culture	(87)
*Non-Ion Channel-Based Mechanosensation*	HA/CD44	Cell adhesion, migration, invasion	Cell Culture	(77)
	IL-33/ST2-R/TNC	Cell invasion	Cell Culture	(76)

HA, hyaluronic acid; TNC, Tenascin-C; ST2-R, ST2 receptor.

Three main classes of mechanosensitive ion channels have been implicated in gliomas: PIEZO, TRP, and ENaC **(** **Table 1** **)**. Of these, mechanotransduction *via* PIEZO and TRP channels have been the most well characterized (85). PIEZO1 specifically in glioblastoma serves as an intermediary for a variety of downstream affects including cytoskeletal remodeling, ECM remodeling as well as directly regulating stemness and aggression of cancer cells (71, 85, 88, 89). Studies have described colocalization of PIEZO1 to regions of cell membrane stress such as those with focal adhesions and signaling *via* the integrin-FAK pathway (71, 88, 89). Different subtypes of TRP channels have been implicated in glioma including TRPC1, TRPC6, and TRPM7 **(** **Table 1** **)** (85, 86, 90–94). Similarly, mechanotransduction of physical microenvironmental stimuli through these channels influences downstream regulation of disease progression through effects key cellular structure and function ranging from motility and migration to proliferation and metabolism (86, 90–94). TRP channels in GBM have been associated with activation of Notch signaling and JAK/STAT signaling pathways (94). Ross et al. also identified a potential role for the ENaC channel which has primarily been shown to regulate cell volume, presumably to facilitate cell motility and migration in complex microenvironments **(** **Table 1** **)** (87). Study of mechanosensitive ion channels has centered largely on those located on the cell membrane, and intracellular or nuclear ion channels in the context of mechanotransduction may represent a new frontier that could improve understanding of downstream signal transduction.

Non-ion channel-based mechanotransduction in glioma is poorly defined and represents an active area of investigation. In recent years, groups have been able to thoroughly characterize a few signal transduction pathways that utilize ligand-mediated, receptor-mediated, and integrin-mediated mechanosensation (45, 76, 77, 79, 95). Other studies in this area have focused on defining downstream components of signaling mediators following the initial mechanosensation event, and this has led to the identification YAP/TAZ, PHIP, and MGAT, among others that play a role in glioma stemness and disease progression; however, a unifying mechanism that considers heterogeneity in mechanical properties of the glioma microenvironment as well genetic and treatment-related drivers of plasticity is lacking and requires further investigation (96–104). Kim et al. describe a ligand-mediated mechanotransduction mechanism in gliomas that leverages the interaction between HA that is overexpressed in the ECM and CD44 **(** **Table 1** **)** (77). In this study, the authors observe a temporal component to mechanotransduction where HA-CD44-mediated signaling and consequent enhanced glioma adhesion appears to occur earlier than integrin-mediated signaling and adhesion in glioma cells cultured in modified hydrogels (77). This suggests that early mechanotransduction may occur *via* an CD44-independent ligand-mediated mechanism whereas the well-described ECM-integrin interactions in glioma may occur later. Temporal cues that define these mechanisms as well as the molecular implications of this phenomenon are unknown. Moreover, this finding adds another potential layer of complexity to mechanotransduction mechanisms in glioma in that our current framework does not clearly define whether temporal heterogeneity exists in this mechanism or the previously described mechanisms and whether this is of functional significance. Several groups have described the mechanotransduction scheme and downstream effects of integrin-mediated signaling (45, 79, 95). TNC has been established as a protein mediator of microenvironment and glioma cell interaction ultimately facilitating and converging on integrin-mediated signaling (70, 76, 105–108). A possible receptor-mediated mechanism for the regulation of mechanosensation has been described by Zhang et al. who show that binding of IL-33 to the ST2 receptor is associated with TNC accumulation and subsequent alteration in the GBM phenotype **(** **Table 1** **)** (76). TNC is an important mediator of mechanical cues in the microenvironment, and these findings suggest that interactions (in this case a receptor-mediated interaction) that alter the availability of such mediators can have implications for mechanotransduction, though further investigation is required to elucidate these potential links.

## Role of Fluid Mechanics in Glioma

### Fluid Shear Stress and Interstitial Fluid Dynamics

Alteration of brain fluid mechanics in patients with glioma, specifically cerebrospinal fluid (CSF) dynamics is not uncommon (109). This can occur secondary to tumor-related obstruction of natural CSF drainage pathways or dysfunction of CSF resorption. Bloodstream-related fluid shear stress and other fluid stresses has been extensively studied in the context of metastatic cancer as well as other disease processes; however, studies characterizing these forces in glioma and examining the impact on the disease process are sparse. Further investigation in this area may provide a more complete view of the stresses at play in the tumor bulk and microenvironment, and early studies suggest that the influence fluid-related stresses may be clinically significant (85, 110–116).

**Figure 2:**
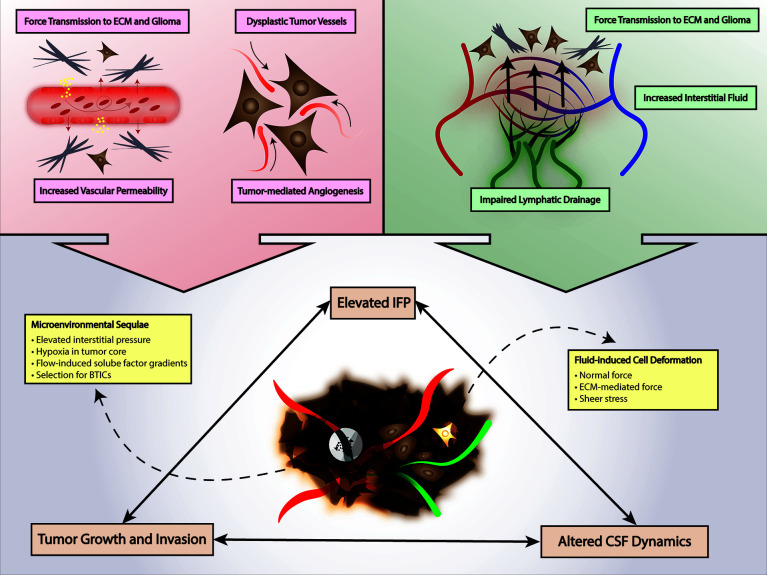
Fluid Mechanics in Glioma. Tumor-mediated angiogenesis and tumor-mediated increased vascular permeability increases interstitial fluid pressure and force transmission to glioma cells and ECM. Lymphatic insufficiency also contributes to increased interstitial fluid pressure. Increased mechanical stimuli from elevated interstitial fluid pressure can promote a tumor environment selective for BTICs and alter CSF dynamics. CSF, cerebrospinal fluid; ECM, extracellular matrix; BTICs, brain tumor-initiating cells; IFP, interstitial fluid pressure.

Interstitial fluid flow refers to fluid flow generally through a 3-dimensional matrix and interstitial fluid pressure (IFP) refers to the biophysical manifestation of the pressure gradient typically between a capillary and a draining lymphatic vessel. In the context of cancer and glioma, an elevated IFP is observed due to increased vessel permeability, i.e., leaky vasculature, secondary to tumor-mediated angiogenesis and dysplastic tumor vessels **(** **Figure 2** **)**. The resultant high IFP has several effects on the tumor and tumor microenvironment. First, elevated IFP is transmitted through the tumor milieu and ECM of the glioma subjecting glioma cells and ECM components to various forces including normal force and shear stress. Much of the current understanding of the sequelae of these fluid-based forces in glioma and in the brain in general are extrapolated from studies in other organ systems and disease contexts; this is in part due to the difficulty to accurately measure these stress forces in a complex microenvironment **(** **Figure 2** **)** (117–119). It is presumable that shear stress-mediated deformation of either the cancer cell cytoarchitecture directly or ECM components promotes changes in stemness, migration, and other features of cancer through mechanotransduction as discussed in previous sections, but this is yet to be investigated comprehensively. Qazi et al. examined the effect of simulated fluid shear stress on the migratory activity of glioma cell lines in a modified Boyden chamber (116). The authors demonstrated that both time of exposure to shear stress and magnitude of shear stress diminished migration in two of three cell lines by 92% and 58%, but the third cell line was not affected by shear stress (116). Quantification of MMP levels showed a concomitant downregulation of active and total MMP with exposure to shear stress that was confirmed with MMP inhibitor assays. Interestingly, the third cell line that was not affected by shear stress also exhibited minimal change in MMP levels. They observed differential migratory activity in the presence of or absence of a TGF-α flow gradient suggesting enhanced cell migration due to a flow-induced chemotaxis—89%, 566%, and 101% enhancement in migratory capacity with TGF-α (116). In similar studies by Li et al. and Namba et al., simulated fluid shear stress applied to U87 glioma cells and BTICs in a microfluidic apparatus produced an increase cellular adhesion strength and differential invasion based on differentiation—less differentiated nestin-positive BTICs tended to invade first under interstitial flow (115, 120). In addition to mechanical effects of fluid shear stress and IFP, these forces in various cancer models including have been shown to produce flow-induced gradients of soluble factors in the microenvironments including chemokines that influence directionality and invasion through chemical signaling **(** **Figure 2** **)** (85, 114, 118, 119, 121). Although initial studies of interstitial fluid flow and IFP posited a radial IFP emanating from the tumor core outward because of the arrangement of leaky vasculature, Spin echo-MRI analysis by Kingsmore et al. of xenograft mouse glioma tumors revealed heterogenous interstitial flow dynamics (110, 111, 122–124). They noted a general trend of outward flow of interstitial fluid but observed significant intratumoral heterogeneity in interstitial flow velocities which correlated with Evans blue assessment of drainage (111). Taken together with the work by Qazi et al. it is evident that fluid dynamics and the response to local fluid dynamics is not uniform but rather heterogenous (111, 113, 116). Further investigation of interstitial fluid dynamics and shear stress in glioma will not only improve our understanding of tumoral and microenvironment heterogeneity but also potentially uncover new therapeutic targets that can be modulated to either slow disease progression or perhaps facilitate enhanced drug delivery to sites of disease (113).

### Lymphatic Flow Dynamics

Lymphatic flow dynamics and drainage effectively link microenvironmental properties such as IFP and interstitial fluid flow with the local immunosuppression that is observed in glioma. In brain tumors such as GBM, draining lymphatic vessels of the tumor are typically compromised, and the tumor milieu is inadequately drained. This produces a dual mechanical and immunological effect in the microenvironment. As a result of compromised lymphatics, interstitial fluid accumulates in certain regions of the microenvironment resulting in elevated IFP with its associated mechanical and chemical signaling as previously discussed **(** **Figure 2** **)** (49, 117, 125–127). Secondly, impaired lymphatic drainage from the tumor simultaneously hinders antigen presentation and immune cell recruitment at peripheral sites as well as preventing migration of immune effector cells into the tumor tissue ultimately producing a function immune escape phenomenon **(** **Figure 1** **)** (49, 125–127). Studies have demonstrated that pharmacologic restoration of these meningeal lymphatic vessels can sensitize GBM to the host immune response and synergize with immunotherapy effectively (117, 125–128). This new class of targeted therapies such as VEGF-C is promising in that it represents a priming of cellular immunotherapy, if successful, can reach infiltrative disease in addition to the primary disease site while concurrently ameliorating malignant mechanical stimuli-induced changes in glioma cells by relieving IFP in areas of impaired lymphatic drainage (49, 125–129).

## Theranostic Opportunities and Modeling

### Therapeutic Targets

Several potential therapeutic targets follow from the present overview of the current understanding of mechanical properties in the glioma microenvironment. Given the crucial role of mechanotransducers such as mechanosensitive ion channels in promoting glioma malignancy, pharmacologic inhibition of signal transduction is attractive. Known inhibitors exist already for some of these channels such as the PIEZO, mechanically gated ion channels; however, non-specificity poses a hurdle as well as the perpetual barrier of achieving effective delivery of any therapeutic to the primary tumor site and invasive BTICs (130–134). Drug pharmacology surrounding the PIEZO channels is still in its infancy, with the majority of studies employing non-specific inhibitors for the purposes of interrogation of channel properties and mechanism of action (130, 133). Groups have identified inhibitors of the PIEZO1 and PIEZO2 channel including the polycation ruthenium red, gadolinium, and the peptide GsMTx-4, but these agents are yet to be tested in translational studies. Activators such as Yoda1, Jedi1, and Jedi have also been described and these agents have been similarly leveraged to elucidate the mechanism of these channels in the glioma microenvironment (71, 133, 135). For example, Chen et al. utilize Yoda1 to further define the relationship between surrounding tissue stiffness and PIEZO1 activity in glioma by showing that beyond a certain level of expression of the channel that is necessary for growth, overactivation of the PIEZO1 channels with Yoda1 do not enhance glioma cell proliferation *in vitro* (71). As *Xiao* outlines in his excellent review of the prospects for future therapeutics targeting PIEZO channels, with increasing recognition of the influence of such ion channels on glioma cell phenotype and disease progression, studies directed towards drug discovery and targeted inactivators of mechanosensitive ion channels for clinical application are forthcoming (133). This is supported by the recent elucidation of the crystal structure of such channels that have paved the path for high-throughput, targeted drug development (136, 137).

In addition to directly inhibiting mechanical signal transduction, another feasible approach is to target the downstream molecular mechanisms and cellular processes that promote disease progression. One such target is autophagy which has been implicated in treatment resistance in various cancers including GBM (138–140). Recent studies have shown that the process of autophagy is altered by mechanical stimuli and signal transduction primarily through two mechanisms: 1) crosstalk between the shared regulatory proteins in autophagy and mechanical signal transduction pathway or 2) competition for molecular substrates utilized in both processes such as cytoskeletal elements (138). Several common pathways have been described in the literature and include the YAP/TAZ axis, JAK-mediated signal transduction, and the expression of EGFR (138, 141, 142). Dupont et al. demonstrate in a series of experiments that the transcriptional regulators YAP/TAZ are required for transducing mechanical cues from the microenvironment and specifically the elasticity of the ECM (96). Using a stem cell model, they demonstrate that altering ECM stiffness and geometry of the growth substrate differentially regulates both proliferation and cell differentiation in a YAP/TAZ-dependent manner (96). Interestingly, YAP/TAZ is also observed to facilitate the fusion of autophagolysosomes and promote autophagic flux *via* a downstream protein target, Armus, in a study by Totaro et al. (101, 143) When this effect was pharmacologically dampened with autophagy inhibitors or through knockdown of autophagy genes, growth of breast cancer cells *in vitro* decreased. The opposite effect was observed when YAP/TAZ was overexpressed, and cancer stem cell features such as plasticity as measured by differentiation potential were enhanced (143). This data suggests that the YAP/TAZ axis may be one of several common pathways between mechanical signal transduction and other aberrant cellular processes in cancer that can be simultaneously targeted (143). Taken together, development of therapeutics that interfere with the cytoprotective effects of autophagy in cancer cells may represent a potential treatment modality and serve as an example for identifying points of convergence between mechanical signal transduction and other known processes in cancer progression amenable to therapeutic targeting (139, 140).

Alternatively, a therapeutic strategy that alters the mechanical properties of the microenvironment, i.e., manipulating solid stress and fluid stress in the tumor milieu, to engineer an anti-glioma environment may be plausible (71, 83, 113, 144, 145). The advantage with such an approach may be the ability to create “smart” or responsive therapies that can either effect mechanical changes locoregionally—in the area of radiographic disease or visible tumor or global changes to target invisible, infiltrative disease (71, 83, 113, 144, 145). Many of the studies discussed in this review as well as other studies examining mechanical properties in the cancer microenvironment have used some type of bioengineering approach to create a microenvironment-mimetic substrate to alter the physical forces that are felt by the cell or tissue of interest. In these studies, modulation of the substrate whether it is a 3D hydrogel or 2D suspension has resulted in elimination of malignant properties. This concept can be theoretically applied as a treatment whereby engineered materials may be implanted into tumor resection cavities after surgical removal of visible tumor. The material or scaffold could be engineered to respond to chemical and physical stimuli from surrounding brain tissue and to alter local physical properties to promote an anti-glioma microenvironment (114, 116, 146–149). This paradigm requires further study and is yet to be developed for clinical application in glioma. Although bioengineering technologies have evolved to meet these needs, this type of approach is hindered by our current rudimentary understanding of biophysical dynamics and their ramifications in brain cancer and in normal brain. Another approach to achieve the same effect is with the use of pharmacologic agents that can alter mechanical properties in the microenvironment such as with the angiotensin inhibitor Losartan. Chauhan et al. show that in models of breast and prostate cancer, administration of Losartan decreases solid stress in the tumor microenvironment by reducing the production of profibrotic components in the ECM such as collagen and hyaluronan (150). The use of Losartan with the intent to target mechanical properties in the context of brain tumors has not been well-studied, but a Phase 2 clinical trial is currently ongoing within this area (NCT03951142). Even in the absence of therapeutics targeted at tissue mechanics, improved understanding of these properties may pave the way for designing adjunctive therapies that can mitigate the biophysical barriers to other treatments such as drug delivery of conventional chemotherapeutics or effective immune cell infiltration of disease sites and successful immunotherapy-based approaches (11, 23, 84, 151).

### Diagnostics

Unique mechanical properties of glioma and its microenvironment provide a basis for the development of novel diagnostics to address two unmet needs in clinical medicine: 1) accurate identification of infiltrative disease in nervous tissue that is radiographically and microscopically occult and 2) characterization disease heterogeneity and plasticity towards predictive analytics for treatment response or prognosis. Fundamental technologies are available as were described in the studies presented in this review such as MRE and other tools for rheological phenotyping, and these have the potential to be adapted and refined as possible intraoperative adjuncts or as supplements to the conventional imaging obtained for patients with GBM to better guide treatment choices (57, 59, 61, 62, 67, 152–154). Briefly, techniques can be categorized based on the substrate assessed—either cells and tissue or more macroscopically a region of the brain **(** **Table 2** **)**. MRE as discussed previously, is now a well-studied imaging technique that can characterize tissue stiffness on a global scale in the brain. In this technique, vibrations through the brain are coupled with magnetic resonance imaging (MRI) sequences to create a landscape of tissue stiffness in the brain (59–62, 85, 155). This is particularly useful when trying to evaluate a mass in the brain as the tissue stiffness within the mass and surrounding regions may offer insights into the tumor type, grade, and propensity for malignant transformation (59, 60, 67). Investigations are ongoing to optimize the imaging protocols for this technique and to develop iterations that can be used at the point-of-care in the operating room. Intraoperative imaging with this technology could provide information regarding prognostication, response to therapy, growth rate in addition to the other intraoperative imaging tools available currently such as Raman spectroscopy, brain mapping, fluorescence-guidance, and optical coherence tomography (156). Further research is needed to establish and validate MRE as a reliable surrogate for such clinical parameters. Ultrasound-based imaging technologies can also be used at the point-of-care, and the two general modalities are traditional ultrasound-based imaging and shear wave elastography **(** **Table 2** **)**. In traditional ultrasound, the ultrasound probe can be placed on the tissue of interest during surgery and an interpretation of physical characteristics is made based on the radiolucency of the area of interest. In shear wave elastography, a device is used to measure the propagation of ultrasonic waves through the region of interest and also measure the displacement of the tissue to calculate physical parameters such as shear modulus (47, 58, 85) **(** **Table 2** **)**. Many methods have been described to study the physical properties of cells and tissue, and these include atom force microscopy, particle-tracking techniques, and measurements of tissue deformation (53–55, 110, 122, 124) **(** **Table 2** **)**. The latter technique consists of lesioning a piece of tissue typically with a needle biopsy, serial slicing, or a single planar cut and subsequently measuring the magnitude of deformation or displacement of the tissue into the lesioned area. Comprehensive reviews of methodologies used to study mechanical properties *in vitro* and *in vivo* can be found elsewhere (47, 85, 155). Advances in bioengineering in the fields of microfluidics, biomimetics, and hydrogel may also enable the development of high-throughput, point-of-care methods to define disease features such as the mechanophenotype that may aid in clinical-decision making (84, 147, 152, 157).

**Table 2:** Representative Methods of Measuring Mechanical Tissue Properties in Glioma.

Method	Substrate	Mechanism	Ref.
MRE	Brain/Tissue	Stiffness map of ROI	(59, 60, 67)
US	Brain/Tissue	Stiffness based on permeability to ultrasonic waves	(85)
SWE	Brain/Tissue	Stiffness based on propagation of ultrasonic waves and tissue displacement	(58)
Needle biopsy	Tissue	Solid stress based on tissue deformation	(54–56, 85)
Serial slices	Tissue	Solid stress based on tissue deformation	(54–56, 85)
Planar cut	Tissue	Solid stress based on tissue deformation	(54–56, 85)
AFM	Tissue/Cell	Stiffness based on force measurement between probe and tissue	(47, 85)
Particle tracking	Tissue/Cell	Live imaging and measurement of particle movement, viscosity measurement	(85)

MRE, Magnetic Resonance Elastography; US, ultrasound; SWE, shear wave elastography.

AFM, Atomic Force Microscopy; ROI, region of interest.

### Preclinical Modeling

Veritable preclinical models of glioma that accurately recapitulate important aspects of the disease are essential for successful clinical translation of innovative therapeutics. Our growing understanding of all the layers of disease heterogeneity and plasticity is now further complicated by heterogeneity and plasticity of the mechanical properties of glioma and its microenvironment (17, 25, 84, 147, 157, 158). Recent work also highlights clinically significant sexual dimorphism in many facets of the glioma disease process, and this feature of glioma has yet to be studied rigorously from the biophysical perspective which will be an important consideration for therapy development (20, 158–160). For example, it is unknown whether sex differences affect solid stress or fluid stress components within a glioma and its microenvironment. Similarly, the influence of other tumor characteristics on the biophysical properties of the tumor microenvironment are also poorly understood. These include factors such as proximity to the cerebrospinal fluid spaces of the brain and the response of tumor to surgical resection and chemoradiation which have been shown to play a role in other processes, but further investigation is needed with regards to mechanical properties (161, 162). Once again, the application of bioengineering to model mechanical features of the microenvironment may be several in dissecting mechanisms of mechanotransduction, mechanoreciprocity, and plasticity. Current models attempt to incorporate different features of heterogeneity in the tumor microenvironment that may impact its mechanical properties such as varying the composition of the ECM constituents as well as the type of model—3D versus 2D (147). Other groups have recreated heterogeneity in stiffness, elasticity, and soluble factor gradients (147, 149, 163, 164). These models will be crucial for effective drug development because they may offer useful information about pharmacokinetic and pharmacodynamic properties of proposed therapeutics as well as uncover additional therapeutic targets or treatment resistance mechanisms. As new variables are identified that can influence the mechanical properties of the microenvironment, the models that attempt to recapitulate these features will likely continue to become more sophisticated (114, 116, 147–149, 165–167).

## Conclusion

The study of mechanical properties in the glioma microenvironment holds considerable promise. Further elucidation of the biophysical features of the microenvironment will enable a more comprehensive understanding of glioma as a disease process, and this may create novel theranostic opportunities while also informing preclinical modeling.

## Author Contributions

AB: conceptualization, investigation, writing - original draft, writing – review and editing, visualization, and management. JD: writing – review and editing. RC: writing – review and editing, and supervision. ST: conceptualization, writing – review and editing and supervision. All authors contributed to the article and approved the submitted version.

## Funding

ST received funding from the National Institutes of Health (R01CA227838) and a pilot project award from the University of Kansas Cancer Center, NCI support grant P30 CA168524.

## Conflict of Interest

The authors declare that the research was conducted in the absence of any commercial or financial relationships that could be construed as a potential conflict of interest.

## Publisher’s Note

All claims expressed in this article are solely those of the authors and do not necessarily represent those of their affiliated organizations, or those of the publisher, the editors and the reviewers. Any product that may be evaluated in this article, or claim that may be made by its manufacturer, is not guaranteed or endorsed by the publisher.
